# Cases Illustrative of the Modus Operandi of Colchicum in the Cure of Gout

**Published:** 1814-11

**Authors:** John Want

**Affiliations:** Surgeon to the Northern Dispensary. North Crescent


*Cases illustrative of the Modus Operandi of Colchicum in the Cure of Gout;* by Mr. Want.

In our last Number, I related two cases of Gout, cured by Colchicum, selected from many others equally successful, because they furnished evidence of the fact, that its curative power is quite unconnected with its purgative quality. I subjoin three more, where the relief was either anterior to the purgative operation, or where no such effect was produced. To strengthen the evidence of these five cases, if it be necessary, I may observe, that the uniform result of my observation, since my first use of the remedy, has been in favour of the inference which I have drawn from them; but, not anticipating the possibility of a dispute concerning facts so obvious, I took no pains to preserve the memoranda with sufficient accuracy for the public tribunal, until these five cases occurred in immediate succession.

As it is my intention to publish very fully on the subject of Gout, and on the efficiency of the various remedies suitable to its different species, I shall not at present enlarge upon the precise application of the Colchicum; but it is incumbent on me to warn my medical brethren, that the utility of a medicine will ever be in proportion to the skilfulness of its administration. If any should fail in the use of it, and failure must inevitably be the case in the hands of some, let not the want of success be hastily laid to the charge of the remedy. If any substance be entitled to the denomination of a specific, it can only be so when properly employed. However valuable it may be, yet, if applied without sufficient discrimination of the circumstances of the case, or attention to the constitution of the patient, it may prove useless; and, in proportion to the strength of its operation on the human body, may manifest a deleterious effect.

CASE I.—Mr. Porter, of Leigh-street, Burton Crescent, applied to me on Wednesday evening, the 12th. He was then labouring under a severe fit of gout in the right hand. For reasons which it is unnecessary to state, the medicine was taken in a very moderate dose, about nine in the evening, and directed to be repeated in eight hours. On the following morning the two doses had been taken, the pain was considerably abated, *though no evacuation had taken place from the bowels*, and it gradually decreased towards evening. I had left directions to repeat the medicine in the same quantity if it were deemed necessary; but, the pain being very trifling, only one dose was given in the evening. On the morrow the pain was extinct, but the medicine had then produced sickness and purging; on Saturday he was quite free from gout. Mr. P. expressed to me his convictions that, had not the disease been attacked by this medicine, the paroxysms would have increased in severity, as he had been accustomed to experience on former attacks.

This gentleman has formerly taken the Eau Medicinale to the extent of 36 bottles, but it produced no sensible effect on him, excepting that of perspiration, though it always removed the paroxysm. The relief from Colchicum was precisely the same, though it was thought not to be so speedy; but this may be accounted for in two ways. In the first place the dose was very small; had a medium quantity been given, a single dose might have been sufficient to remove the pain in the usual time, though it might still be short of that which could operate on the bowels; or the disease, from its longer continuance, or other circumstances, might require a fuller dose for its cure.

CASE II.—Mr. Dingell, landlord of the public-house at the corner of Tavistock-place, Little Coram-street, sent to me on Tuesday evening. The pain was confined to the foot and ancle; he took the Colchicum immediately. On the following morning I called, but did not see him; I believe he was from home; but Mrs. D. assured me he was quite free from pain, and that *no operation on the stomach or bowels had been occasioned by the medicine.*

A circumstance in the case of this patient may deserve to

4. be be recorded. In the intervals of the paroxysms a large and hard tumour is perceived in the ham; but, on their invasion, it invariably begins to soften and diminish, until it is scarcely perceptible.

In my paper published in the Number for September 1811, I advocate the cause of the humoral pathology, in opposition to Cullen and his disciples. Is not this tumour one among many other proofs of the existence of morbific matter?

CASE III.—Ann Ellis, 23, Chapel-place, Little Coram-street, came under my care at the Northern Dispensary for a severe scald. She was unhappily labouring at the same time under gout, to which she has been these five years subject. The last time she was laid up with it, *she took medicines without effect for the space of three months.* I saw her, and gave her the medicine, which she took at ten o'clock on Friday evening, the 14th. The pain was fixed in the hip and toe, had been in the elbow, and was coming on in the hands; in the toe and the elbow, it was attended with great swelling; this is never seen in the hip, but the pain is excruciating there. At two o'clock, on getting out of bed, she found the toe was nearly well, and was able to stand; the pain continued a little in the hip; in the morning, at ten, it was quite gone. At the time of noting this account (Sunday morning) she is quite well, and has walked to my house in the Crescent without any difficulty; the pain in the toe was considerably relieved *before any evacuation from the bowels:* she says, if it had not been so she could not have stood upon it. The only motion in the night was after *the relief from pain in the toe*, and she had no other till she took her breakfast at ten o'clock in the morning, when she had five more evacuations, which were watery. The operation of the medicine she considered to be mild.

JOHN WANT,
*Surgeon to the Northern Dispensary.*

*North Crescent, Oct.* 1814.

